# Publisher Correction: Multi-orbital charge transfer at highly oriented organic/metal interfaces

**DOI:** 10.1038/s41467-017-01480-w

**Published:** 2017-11-30

**Authors:** Giovanni Zamborlini, Daniel Lüftner, Zhijing Feng, Bernd Kollmann, Peter Puschnig, Carlo Dri, Mirko Panighel, Giovanni Di Santo, Andrea Goldoni, Giovanni Comelli, Matteo Jugovac, Vitaliy Feyer, Claus Michael Schneider

**Affiliations:** 1 Peter Grünberg Institute (PGI-6), Forschungszentrum Jülich GmbH, D-52425 Jülich, Germany; 2 Institut für Physik, Karl-Franzens-Universität Graz, NAWI Graz, 8010 Graz, Austria; 3 Department of Physics, University of Trieste, Via A. Valerio 2, 34127 Trieste, Italy; 4 IOM-CNR Laboratorio TASC, S.S. 14 km 163.5 in AREA Science Park, Basovizza, I-34149 Trieste, Italy; 5 Elettra—Sincrotrone Trieste, S.S. 14 km 163.5 in AREA Science Park, Basovizza, I-34149 Trieste, Italy; 6 Fakultät f. Physik and Center for Nanointegration Duisburg-Essen (CENIDE), Universität Duisburg-Essen, D-47048 Duisburg, Germany

*Nature Communications* **8**:335 10.1038/S41467-017-00402-0; Article published online 25 August 2017

The original version of this Article contained an error in the spelling of the author Claus Michael Schneider, which was incorrectly given as Claus Michael Schneidery. This has now been corrected in both the PDF and HTML versions of the Article.

