# Supplementary material for: Multi-orbital charge transfer at highly oriented organic/metal interfaces
Source: Nat Commun. 2017 Aug 25;8:335. doi: 10.1038/s41467-017-00402-0 (PMC5570996; doi:10.1038/s41467-017-00402-0)
Supplement: Supplementary file 2 — Supplementary Information [file 41467_2017_402_MOESM2_ESM.pdf]

File name: Supplementary Information
Description: Supplementary Figures, Supplementary Notes and Supplementary References.

File name: Peer Review File
Description:

Supplementary Note 1

The Ni-TPP appearance in the STM images does not noticeably change for biases in the [-2.0;+1.0] V range, while the phenyl features start to blur when approaching the +2.0 V bias (see Supplementary Figure 1).

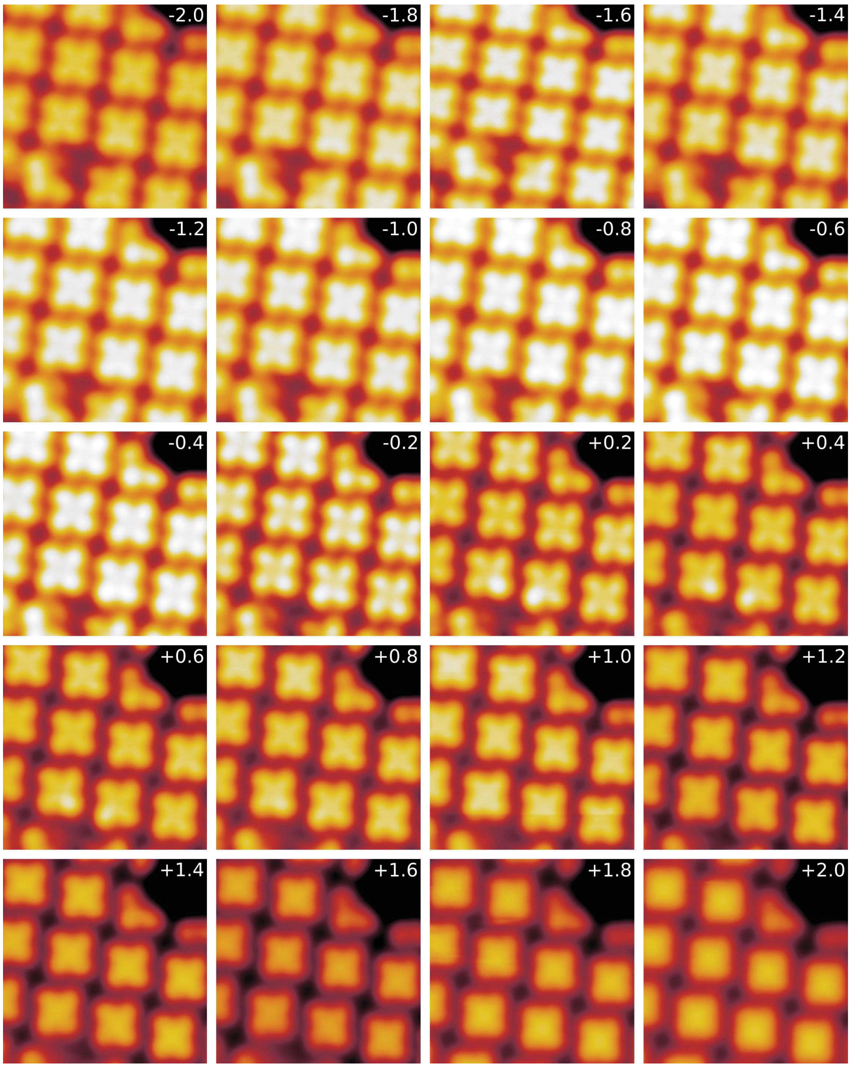

Supplementary Figure 1: Series of STM images of the same region on the surface, acquired at different bias voltages (correspondent bias written at the top-right of each images). For all images, STM parameters: $I_{\mathrm{t}} = 0.5$ nA, 4.4x4.4 nm^2.

The absence of significant contrast changes on the macrocycle region, when varying the bias voltage in the whole range, suggests that even when the tip is placed above the TPP

center, the states related to the macrocycle cannot be deconvolved from the contribution of the phenyl ligands. This is in agreement with the simulated PDOS reported in the manuscript (see Figure 4c). Indeed, in the [-3.0;+2.0] eV range, the projected DOS on phenyl groups has a rather featureless plateau, whereas, beyond this range it increases very rapidly.

At +2.4 eV the DFT simulated image is almost identical to the experimental one (see Supplementary Figure 2).

(a) Simulated Image (+2.4 V) (b) Experimental Image

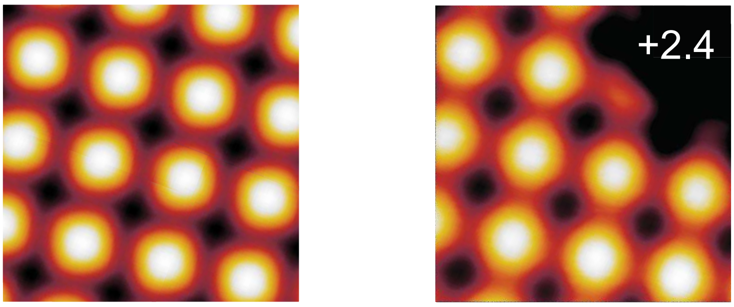

Supplementary Figure 2: Comparison of a simulated image at +2.4 eV (left) with a STM image at a bias of +2.4 V (right), STM image parameters: I_t = 0.5 nA, 4.4x4.4 nm^2.

Supplementary Note 2

In order to further analyze the work function changes upon adsorption of the molecular layer, we have calculated the plane averaged charge density differences $\Delta\rho$ as the difference between the charge density of the full system ρ_f and the sum of the charge densities of the two separate systems, *i.e.* the freestanding molecular layer ρ_m and the substrate slab ρ_s:

$$\Delta\rho = \rho_f(r) - (\rho_m(r) + \rho_s(r)) \tag{1}$$

The corresponding results are shown in Supplementary Figure 3a. Here, red areas correspond to the regions of charge accumulation, while blue areas show regions of charge depletion. One can see, that a series of rather large dipoles appear upon adsorption in the z direction, parallel to the surface normal. There are two effects which create dipoles of opposite sign and which compensate the resulting changes in work function. First the charge spill-out is pushed into the metal (push back effect), which induces a dipole that reduces the work function and second charge transfer from the metal to the molecule which enhances the work function. The changes in the electrostatic potential, *i.e.* the change in work function, due to these dipoles, can be calculated by solving the one dimensional Poisson equation for the charge density difference, integrating it twice along the z coordinate. The result is shown as an orange line and emphasizes that in total the dipoles induced by the adsorption reduces the work function and hence that the push back effect dominates. The reduction of the work function takes place despite the rather large charge transfer to the molecule and can be understood due to the small distance between adsorbate and metallic substrate. Work function changes of the system, upon Ni-TPP adsorption, have been measured using our PEEM set-up. All changes were referred to the value for the clean copper work function. The work function difference between the bare Cu(100) and Ni-TPP/Cu(100) has been defined as the difference of the raised edge of the secondary electron signal of photo-emission spectra for the two systems, as shown in Supplementary Figure 3b. The Ni-TPP, upon adsorption, lowers

the work function of the overall system by ∼0.80 eV, which is in agreement with the DFT predictions.

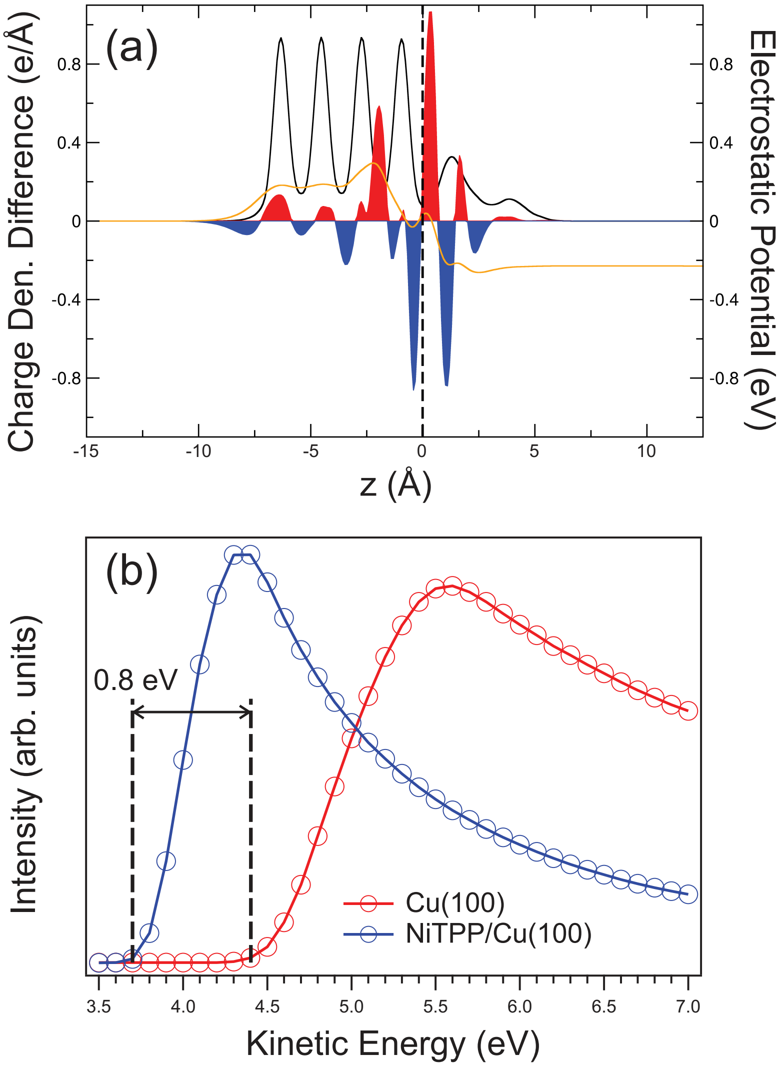

Supplementary Figure 3: (a) Plane-averaged charge density difference (red-blue curve, left axis) and electrostatic potential (orange curve, right axis) as a function of the z direction, perpendicular to the substrate. In order to indicate the position of the metallic layers and the molecular layer, the plane averaged charge density of the full system is shown as a black line in arbitrary units. The surface is located at ∼ 0 Å (dashed line) (b) Photoemission spectrum at the secondary electron onset for bare copper (red curve) and NiTPP/Cu(100) (blue curve), taken at $h\nu = 27$ eV.

Supplementary Note 3

For clarity, in Supplementary Figure 4, we report the molecular orbitals (MOs) from HOMO-3 up to LUMO+3 with the correspondent $|FT|^2$. MOs are calculated starting from the Kohn-Sham eigenstates of a gas phase molecule, and then a 3D Fourier transform (FT) of each considered orbital is performed.[1] Subsequently, the 2D map is obtained by cutting the 3D FT at constant k value, *i.e.* at constant electron kinetic energy. This method is based on the assumption that the final state of the photo-emitted electron from the i-*th* molecular orbital can be approximated by a plane wave.[1]

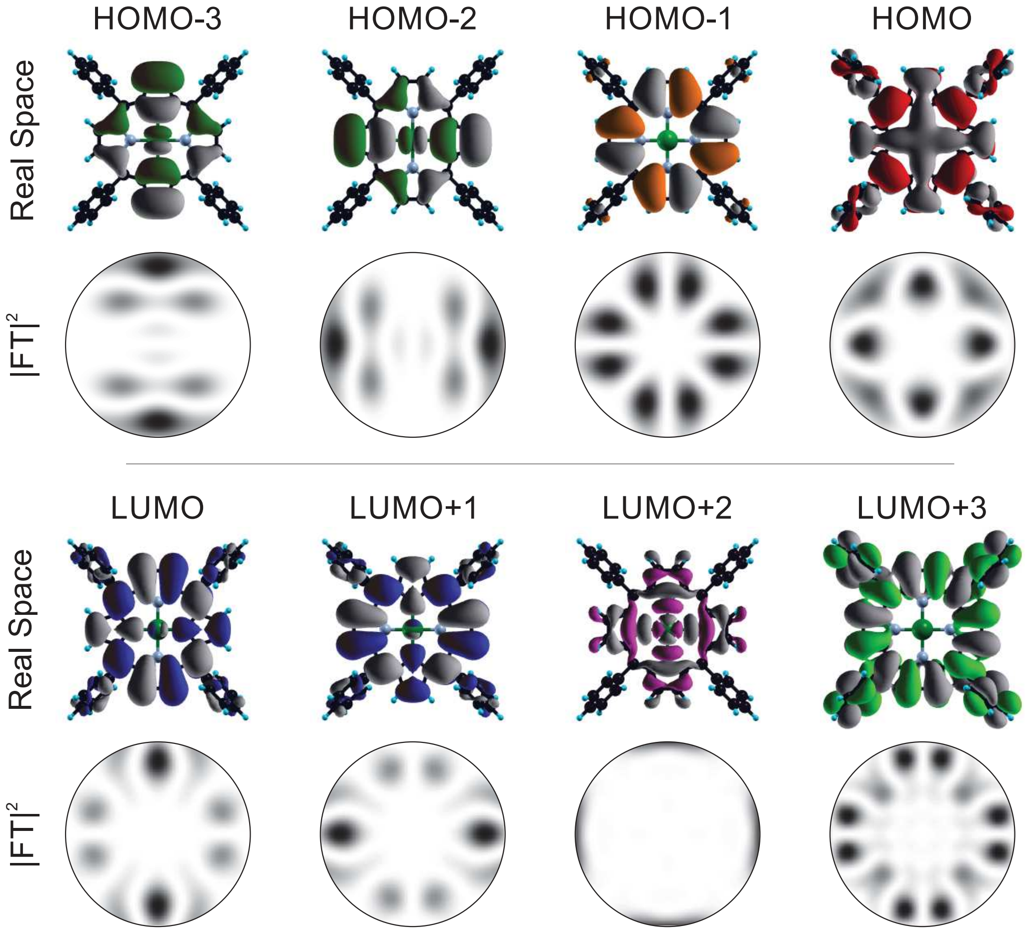

Supplementary Figure 4: Calculated real space molecular orbitals for the gas-phase NiTPP, from HOMO-3 up to LUMO+3, with the correspondent $|FT|^2$ below. The k range in the $|FT|^2$s is: $k_x, k_y \in [-2, +2]$ Å^{-1}.

The Kohn-Sham eigenstates have been calculated by the NWCHEM[2] DFT code, utilizing the range separated Heyd-Scuseria-Ernzerhof (HSE) hybrid functional[3] for exchange-correlation effects. We would like also to point out that the so obtained $|\mathrm{FT}|^2$ cannot be directly compared to the μ-ARPES maps. This operation requires an additional step: contributions from the two rotational domains have to be taken into account.

Supplementary References

(1) Puschnig, P. *et al.* Reconstruction of molecular orbital densities from photoemission data. *Science* **326**, 702-706 (2009).

(2) Valiev, M. *et al.* NWChem: a comprehensive and scalable open-source solution for large scale molecular simulations. *Comput. Phys. Commun.* **181**, 1477-1489 (2010).

(3) Heyd, J., Scuseria, G. E. & Ernzerhof, M. Erratum: Hybrid functionals based on a screened Coulomb potential [J. Chem. Phys. 118, 8207 (2003)]. *J. Chem. Phys.* **124**, 219906 (2006).
